# An integrated remote sensing and GIS approach for monitoring areas affected by selective logging: A case study in northern Mato Grosso, Brazilian Amazon

**DOI:** 10.1016/j.jag.2017.05.001

**Published:** 2017-09

**Authors:** Rosana Cristina Grecchi, René Beuchle, Yosio Edemir Shimabukuro, Luiz E.O.C. Aragão, Egidio Arai, Dario Simonetti, Frédéric Achard

**Affiliations:** a European Commission, Joint Research Centre (JRC), Directorate D – Sustainable Resources, Bio-Economy Unit, Ispra (VA), Italy; b Brazilian National Institute for Space Research (INPE), São José dos Campos (SP), Brazil

**Keywords:** Brazilian Amazon, Forest degradation, Selective logging, Remote sensing, Landsat

## Abstract

Forest cover disturbances due to processes such as logging and forest fires are a widespread issue especially in the tropics, and have heavily affected forest biomass and functioning in the Brazilian Amazon in the past decades. Satellite remote sensing has played a key role for assessing logging activities in this region; however, there are still remaining challenges regarding the quantification and monitoring of these processes affecting forested lands. In this study, we propose a new method for monitoring areas affected by selective logging in one of the hotspots of Mato Grosso state in the Brazilian Amazon, based on a combination of object-based and pixel-based classification approaches applied on remote sensing data. Logging intensity and changes over time are assessed within grid cells of 300 m × 300 m spatial resolution. Our method encompassed three main steps: (1) mapping forest/non-forest areas through an object-based classification approach applied to a temporal series of Landsat images during the period 2000–2015, (2) mapping yearly logging activities from soil fraction images on the same Landsat data series, and (3) integrating information from previous steps within a regular grid-cell of 300 m × 300 m in order to monitor disturbance intensities over this 15-years period. The overall accuracy of the baseline forest/non-forest mask (year 2000) and of the undisturbed vs disturbed forest (for selected years) were 93% and 84% respectively. Our results indicate that annual forest disturbance rates, mainly due to logging activities, were higher than annual deforestation rates during the whole period of study. The deforested areas correspond to circa 25% of the areas affected by forest disturbances. Deforestation rates were highest from 2001 to 2005 and then decreased considerably after 2006. In contrast, the annual forest disturbance rates show high temporal variability with a slow decrease over the 15-year period, resulting in a significant increase of the ratio between disturbed and deforested areas. Although the majority of the areas, which have been affected by selective logging during the period 2000–2014, were not deforested by 2015, more than 70% of the deforested areas in 2015 had been at least once identified as disturbed forest during that period.

## Highlights

• A new approach for monitoring areas affected by selective logging is proposed.
• We use combination of object-based and pixel-based classification approaches.
• Logging intensity and changes over time are assessed within grid cells.
• Changes in forest cover are assessed consistently through time.

## 1 Introduction

Forest degradation is a pervasive environmental issue, especially in the tropics, which together with deforestation have important impacts on biodiversity and human well-being, and significant contribution to greenhouse gas emissions (Thompson et al., 2013). In the Brazilian Amazon (BA), the world's largest expanse of tropical forest, forest areas have been significantly impacted by forest degradation due to different anthropogenic forest disturbance processes such as selective logging, forest fires and forest fragmentation (Souza, 2013). It has been estimated that the area of forest affected by selective logging in the BA is as high as the area of deforestation, representing additional harm not addressed in deforestation studies (Asner et al., 2005). In fact, degraded forests (either by fire or logging) identified by INPE's DEGRAD system (INPE, 2008) were almost twice the area of deforestation in the period of 2007–2013 (Aguiar et al., 2016).

Despite the economic importance of the logging industry (e.g. generating revenues and jobs) and the potential it has as renewable resource, logging activity in the Brazilian Amazon has resulted in substantial ecological damage due to the high volume of extraction, poor management practices, and very often illegal (unplanned) activities (Asner et al., 2009). Mapping forest areas affected by these anthropogenic disturbance processes (like selective logging) is the first step to understand forest changes leading to degradation. Plenty of definitions for forest degradation and degraded forest have been proposed (Lund, 2009); however, a common definition is still under debate. In this research, we are mainly concerned with human-induced disturbances (e.g. tree harvesting, road construction) causing a change process that can negatively affect the forest functioning and lead to long-term forest degradation (FAO, 2011).

Knowing where and how changes are happening allows for tackling the problem, supporting planning and protective measures and proposing reduction targets, especially within the context of incentive mechanisms such as REDD+ (Reduction of Emissions from Deforestation and Forest Degradation). Consequently, reliable information about the extent of forest cover disturbances is highly needed (FAO, 2011).

Remote sensing products have been a key source of information for monitoring land cover changes in the past decades (Lunetta et al., 2002). Moreover, these products can be considered as the single feasible way of consistently monitor changes in forest cover over time for large geographical regions (Shimabukuro et al., 2014). In the Brazilian Amazon, according to Souza (2013), remote sensing has been used for mapping selective logging, from local to regional scales, and the approaches range from visual interpretation to automated techniques. The author also highlights the differences in possible mapping goals, for instance mapping the total area affected by logging (which includes canopy damaged areas, cleared areas or logging infrastructure, and portions of intact forest), or mapping damaged areas only. Examples of studies using visual interpretation include Watrin and Rocha (1992) and Stone and Lefebvre (1998). A technique often applied in the BA is the Spectral Mixture Analysis (SMA), which is used to enhance the detection of selective logging areas and to deal with the problem of heterogeneous land cover in these environments, especially for medium resolution images such as Landsat (Souza, 2013). Examples for the usage of SMA include Souza and Barreto (2000), Anwar and Stein (2012), and Asner et al. (2004). Souza et al. (2005) proposed a new index based on SMA called Normalized Difference Fraction Index (NDFI), for detecting forest canopy damage due to selective logging or forest fires. This index has been used e.g. by the Mato Grosso State government for mapping logging areas for the 2012–2013 period based on visual interpretation of the NDFI images (SEMA-MT, 2015). Other studies have applied SMA in combination with GIS techniques (buffering), such as Souza and Barreto (2000), Monteiro et al. (2003) and Matricardi et al. (2005). Asner et al., 2005, Asner et al., 2006 used the Carnegie Landsat Analysis System (CLAS) an automated method based on SMA and pattern recognition for assessing forest disturbances for large areas of the Brazilian Amazon. A recent study by Pinheiro et al. (2016) used data mining techniques and cell approach for classifying forest degradation patterns based on a defined forest degradation typology and landscape metrics. Object-based image analysis is a method, which has rarely been applied in this region for mapping selective logging (e.g. Monteiro et al., 2007). The Brazilian National Institute for Space Research (INPE) has estimated forest degradation for the Brazilian Amazon since 2007 through a visual interpretation procedure as part of the DEGRAD project (Forest Degradation Mapping in the Brazilian Amazonia); however mainly areas heavily disturbed (by selective logging and/or fire) with a tendency to be cleared were considered (INPE, 2008). Most of the remote sensing based research, regarding the mapping of logged forests in this region, used Landsat imagery, which is indicated currently as the most feasible data, considering that it is cost-free and regularly acquired (Souza, 2013). Finer resolution images (e.g. IKONOS) can enhance the detection of selective logging but have considerable cost and computational constraints (Asner et al., 2009). It is expected that the availability of time series of finer spatial resolution data such as Sentinel-2 (10 m × 10 m spatial resolution) will improve the assessment of logging areas. However, even using visual interpretation and finer resolution images, defining the boundaries of logged areas is not trivial (Souza, 2013).

Despite many initiatives for assessing selective logging in the BA, mapping the areal extent of logged forests using remote sensing imagery is still reported as a challenge (Asner et al., 2009) especially because of (1) the highly dynamic spatial–temporal patterns of logging activities, which can be detected by remote sensing only for a limited amount of time due to rapid canopy closure after the logging events (Asner et al., 2009), (2) the complexity of these forest environments, encompassing green vegetation, dead trees, bark, bare soil (Souza, 2013), (3) the occurrence of logging associated with other processes, such as forest fires (Souza, 2013), and (4) the fact that logging activities show different intensities and patterns, depending on logging techniques used and on how the activity is performed (planned/unplanned), amongst others. Moreover, the lack of a consensus definition has hampered forest degradation mapping and monitoring in general (Thompson et al., 2013).

In this context, our main goal was to investigate the annual changes in forest areas due to forest disturbance processes (mainly selective logging) in the Brazilian Amazon from a Landsat multi-temporal dataset over a 15-year period. For this aim, we propose a new method based on fraction images and GIS techniques, which allows to map and monitor forest disturbances due to selective logging. Our method can concomitantly assess deforestation and could be later adapted to integrate other forest disturbance processes such as fire.

## 2 Study area

**Fig. 1:**
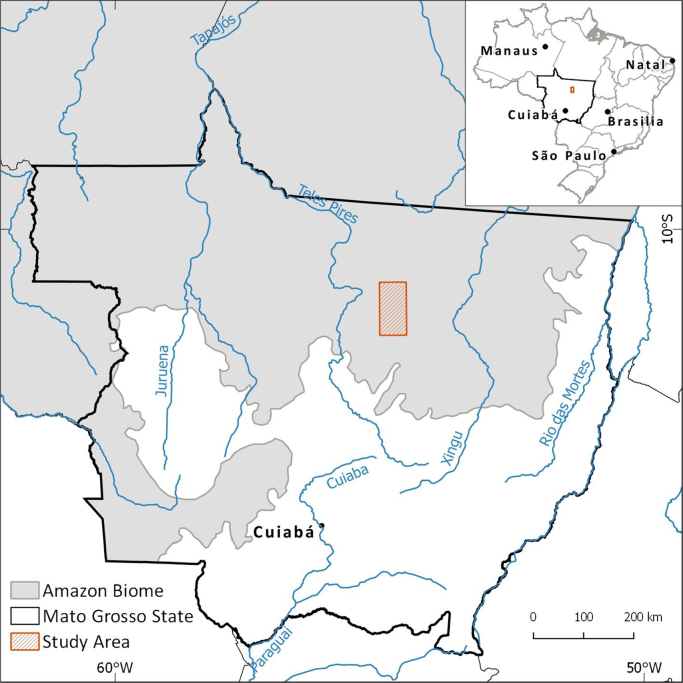
Location of the study area in the Brazilian Amazon biome and Mato Grosso State.

The study area (Fig. 1) is located in the central-northern part of Mato Grosso State, within the Amazon biome (IBGE, 2004). The size of this area is c. 55 km × 111 km and corresponds to a subset of a Landsat scene (path/row 226–68). For investigations of forest cover changes, Mato Grosso is a key state of the Brazilian Amazon with very high deforestation rates and high occurrence of areas affected by forest disturbances (INPE, 2008). The selected study area is among the main timber production centers of Mato Grosso and encompasses parts of municipalities which hold very high occurrence of illegal logging for the 2012–2013 period such as Marcelândia, União do Sul e Santa Carmem (SEMA-MT, 2015). The forests in this area are described as transitional forest or ecotone areas between ombrophilous and seasonal forests (IBGE, 2004).

## 3 Materials and methods

### 3.1 Datasets/satellite imagery

Yearly cloud-free Landsat images were selected during the period 2000–2015 for developing the methodology, together with auxiliary datasets, which were available for some of the years taken into consideration. It resulted in a temporal dataset of 15 Landsat images (TM, ETM+, and OLI sensors) (Table 1). Due to the rapid forest cover changes related to logging activities, an annual mapping of areas affected by selective logging is recommended (GOFC-GOLD, 2013). Images were converted to top of atmosphere (TOA) reflectance using the JRC Impact toolbox (Simonetti et al., 2015).

**Table 1:** Satellite imagery and auxiliary data used.

Satellite imagery		Auxiliary data
Sensor	Dates	• PRODES – Amazon Deforestation Monitoring Project (INPE, 2008) – deforestation information (2000–2015)
LANDSAT ETM+	18-06-2000	• DEGRAD – Forest Degradation Mapping in the Brazilian Amazon (INPE, 2008) – forest degradation information (2007–2013)
LANDSAT ETM+	08-08-2001	• GFC – Global forest change project (Hansen et al., 2013) – tree cover 2000 and forest cover loss (2001–2014)
LANDSAT ETM+	10-07-2002	• RAPIDEYE Image 2013-09-23
LANDSAT TM	21-07-2003	• Sentinel 2 image – 2016
LANDSAT TM	23-07-2004	
LANDSAT TM	26-07-2005	
LANDSAT TM	27-06-2006	
LANDSAT TM	16-07-2007	
LANDSAT TM	18-07-2008	
LANDSAT TM	22-08-2009	
LANDSAT TM	24-07-2010	
LANDSAT TM	12-08-2011	
LANDSAT OLI	16-07-2013	
LANDSAT OLI	04-08-2014	
LANDSAT OLI	22-07-2015	

Most of the selected images are spanning from June and July, which helped to focus on selective logging and to avoid the impacts of burned areas from the current year, as most of the fires in the region occur between August and September (Shimabukuro et al., 2015). Moreover, we observed a general predominance of selective logging over burned forests in this area. However, old burned areas could not be separated from selective logging (burned areas and logging can also occur together), thus they were mapped as “disturbance”, as they predominantly correspond to heavily disturbed forest areas.

### 3.2 Methodological approach

We applied a stepwise approach to identify forest areas affected by selective logging in this region, consisting in:

• mapping forest, “non-forest” (crops, pastures, roads, water, urban), and new deforested areas for each year using an object-based classification approach;
• extracting logging “evidences” (e.g. logging decks, logging roads, tree-fall gaps) from data gained through a Linear Spectral Mixture Analysis – LSMA (Shimabukuro and Smith, 1991);
• assessing logging intensity and logging dynamics over time through a GIS analysis based on regular grid-cells of 300 m × 300 m.

This approach was chosen for the following reasons:

• pure pixel-based approaches were leading to the partial identification of logged areas as small clearings (i.e. as deforestation instead of forest disturbances);
• an object-based approach enables a good delineation of forest and non-forest areas and the inclusion of small clearings (due to logging) as part of the forest patches;
• soil fraction images can be used to highlight logging infrastructure considering the issue of mixed land cover at 30 m spatial resolution (Souza, 2013);
• the intense spatial-temporal dynamics of logging activities makes their assessment over time more challenging.

**Fig. 2:**
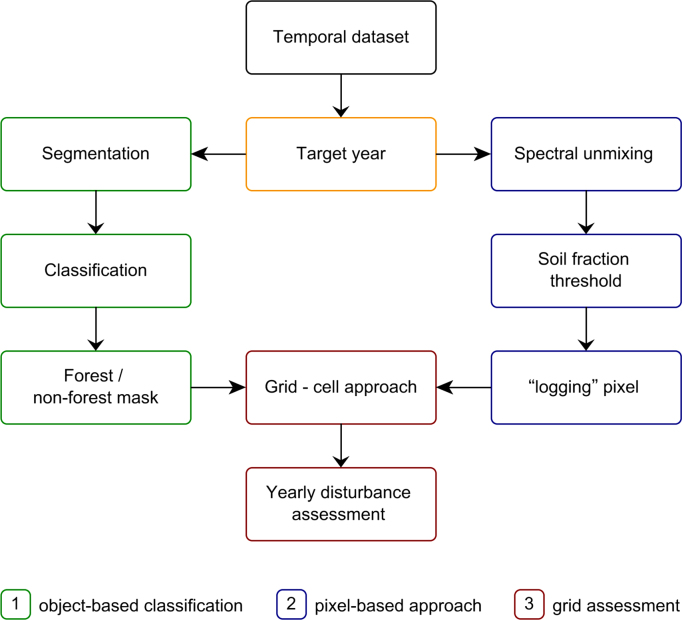
Summary flowchart of the proposed methodology including three main parts: (1) building a forest mask from an object-based classification approach, (2) extracting logging areas from soil fraction images and (3) integrating information using a grid-cell approach for reporting on forest disturbance intensity (red). (For interpretation of the references to color in this figure legend, the reader is referred to the web version of this article.)

A summary of the methodology is presented in Fig. 2 and described in the following sections.

#### 3.2.1 Building forest masks

**Fig. 3:**
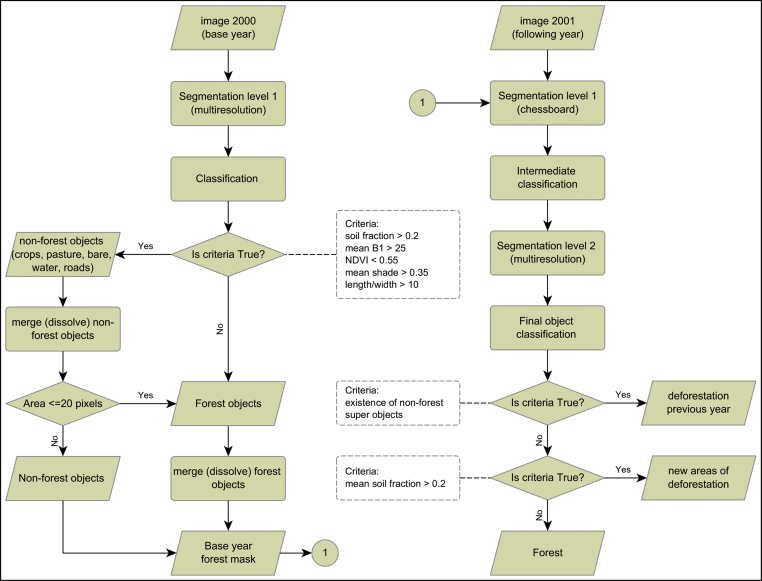
Workflow of the procedure for building the forest mask of the base year and its integration into the classification process of the following year.

Through an adaptation of the methodology used by Shimabukuro et al. (2014), the first step of our approach consisted in building forest masks, initially for the base year and then updating it with new deforested areas for the following years of the temporal series. We developed an object-based classification approach using eCognition^®^ software (version eCognition Developer 9) with two main process trees. The first process tree was built to produce the initial forest mask and consisted in the segmentation of the base year image (year 2000) and the classification of image objects into forest and non-forest classes. For the classification of image objects, we followed an interactive and sequential procedure using a decision tree approach of selected attributes able to identify firstly all non-forest areas such as crops, main roads, water bodies. Thereafter the remaining areas were classified as forest. At this stage, we aimed at keeping the logging features (e.g. logging decks or forest canopy gaps) within the forest patches. An area criteria was applied in the end of the non-forest mapping in order to correct eventual logging areas classified as non-forest. Building a forest mask through an object-based approach was an essential step to avoid mapping logging evidences as small clearings. The second process tree was designed to take into consideration the forest mask from previous year (thus keeping the segmentation consistent over time) and to update it with the new deforested areas. For this second process tree, we applied a two-level segmentation in order to (1) integrate the forest mask from previous year and (2) to identify/map new deforested areas. A summary of segmentation parameters is presented in Table 2, and the steps of the proposed object-based approach depicted in Fig. 3. It has to be noticed that we did not assess regrowth from deforested areas in our study, as our focus was to assess changes within the forest areas. The forest masks of this study are available at: http://forobs.jrc.ec.europa.eu/products/selective_logging_mt/.

**Table 2:** Segmentation parameters used in this research.

Forest mask	Segmentation algorithm	Scale	Color/shape smoothness/compactness	Layers	Objectives
Base year	Multiresolution	10	0.8/0.2	Bands 3, 4, 5	Identify forest and non-forest areas
			0.5/0.5		
Other years	Chessboard	1000	–	Thematic (previous years’ forest mask)	Incorporate forest mask from previous year
	Multiresolution	50	0.9/0.1	Bands 3, 4, 5	Identify new clearings
			0.5/0.5		

#### 3.2.2 Mapping disturbances within the forest

We applied the LSMA (Shimabukuro and Smith, 1991) in order to retrieve information at a sub-pixel level and enhance logging features, considering the heterogeneity of land cover in these environments at the spatial resolution of c. 0.1 ha (30 m × 30 m). The unmixing approach assumes that a pixel value results from the combination of the reflectance of distinct components (endmembers) to estimate the proportion of each component within a pixel (Shimabukuro et al., 2009, Dennison and Roberts, 2003). Endmembers representing soil, vegetation and water/shade were selected from the images to be classified. A radiometric normalization based on pseudo-invariant features (Bodart et al., 2011, Achard et al., 2014) was applied to reduce the spectral differences across the temporal series. Both spectral unmixing and forest normalization were batch-processed using the JRC Impact Toolbox.

**Fig. 4:**
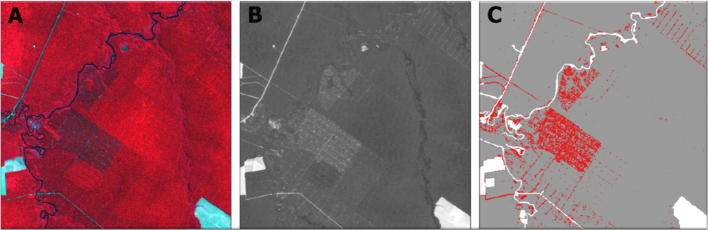
Steps of extracting logging evidences, example of the image of year 2009: (A) Landsat image, (B) soil fraction image, and (C) logged areas in red. (For interpretation of the references to color in this figure legend, the reader is referred to the web version of this article.)

Fraction images containing the information on endmembers proportions were generated and, subsequently, logging areas extracted from the soil fraction images. An empirically defined threshold of 10% was applied to all soil fraction images in order to identify logged areas (see Fig. 4). The threshold definition was assisted by the visual assessment of temporal profiles of soil fraction images for key areas (e.g. undisturbed forest, logged forest and cleared areas). The threshold was defined by testing soil fraction values within forest areas with diverse logging intensities to allow the mapping of most of the logging infrastructure (e.g. logging decks and logging roads) and canopy openings (e.g. heavily logged areas and burned areas). The 10% threshold has been used by Souza et al. (2005) for a similar purpose based on field work information on log landings, thus corroborating the use of this threshold value.

#### 3.2.3 Assessing disturbances: grid-cell approach

Assessing the geographical extent of the areas affected by logging is not straightforward. In this research, for reporting the areas affected by selective logging we use a regular grid with cells of 300 m × 300 m. The use of such grid is needed as logging is highly dynamic in space and time. The average distance between logging decks in this area (between 200 m and 250 m) was taken into consideration for the choice of the grid cell size, as well as the need to consider contextual information. The chosen grid cell size is close to the recommendation of a radius of 180 m to assess the area affected by logging made in other studies (Souza and Barreto, 2000, Matricardi et al., 2005).

**Fig. 5:**
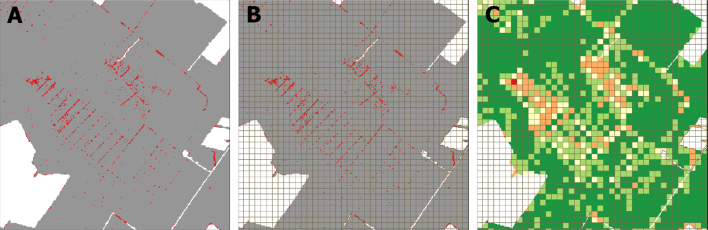
Example of (A) map of forest, non-forest and logging, (B) application of grid cells, and (C) resulting map of logging intensity (dark green: undisturbed forest, light green: very low disturbance, beige: low disturbance, orange: medium disturbance, red: high disturbance). (For interpretation of the references to color in this figure legend, the reader is referred to the web version of this article.)

Based on a combination of the forest mask produced in step 1 with the map of logging areas from step 2, for each grid-cell the percentages of forest, deforested areas and “logging” pixels are computed for each single year. The pixels of each category (forest, deforested and “disturbed”) are counted within each grid-cell. The disturbance intensity is calculated considering the amount of “logging pixels” in relation to the total forest area and categorized as follows: very low intensity (1–5%), low (5–10%), medium (10–50%), and high (>50%) (Fig. 5).

**Fig. 6:**
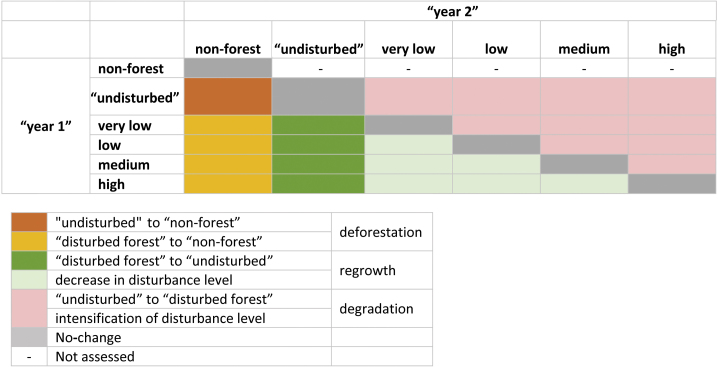
Example of calculation of change trajectories for a time interval T1–T2.

We then produce areal statistics, yearly maps of disturbance intensity and transition matrices between land cover classes for annual time intervals (e.g. 2000–2001, 2001–2002, etc.). We also calculate the annual rates of deforestation and forest disturbance as ratios between yearly new deforestation and newly logged areas divided by the total forest area at the initial year (Fig. 6). Moreover, we assessed the number of times a grid-cell was detected as “disturbed” during the 15-years period.

#### 3.2.4 Accuracy assessment

Ideally the accuracy assessment of land cover maps produced using Landsat-type imagery should be based on field data or higher resolution imagery (Liu, 2008, Strahler et al., 2006). However, these reference datasets can be very difficult to obtain, especially when historical information is needed. Moreover, in the case of selective logging, the reference dataset should be of a close date for comparison due to the rapid changes of logging activities. Thus, for assessing the accuracy of our results, we opted to build a reference dataset thought visual interpretation by an independent expert, based on the same imagery used for the production of the maps. This showed to be the only feasible way of assessing historical information on logging dynamics. The approach of using independent expert visual interpretation has already been employed (e.g. Beuchle et al., 2015); it can provide a quantitative indication of the potential errors.

The accuracy assessment encompassed two main parts (1) validating the initial forest mask, which served as the basis for updating deforestation for the following years and (2) validating information on forest disturbances. The baseline forest/non-forest mask (year 2000) was assessed based on 300 sample points randomly distributed (150 on the forest and 150 on non-forest areas), interpreted by the independent interpreter. We also compared two existing datasets, the deforestation maps from the PRODES project (INPE, 2008) and the Global Forest Cover Change (GFC) product (Hansen et al., 2013), with this independent reference dataset. For this comparison, the PRODES maps were edited considering all deforested areas up to year 2000 as non-forest and the remaining areas as forest. For the GFC product (year 2000) we considered as forest areas pixels with tree cover >30%.

The second part of the assessment consisted in looking only within our forest class and assessing forest disturbance information based on 900 grid-cell units, distributed in four random years (2003, 2007, 2011, and 2015), and interpreted by an independent expert. A stratified random sampling approach (Jensen, 2005), considering 4 classes (‘undisturbed’, ‘low’, ‘medium’, ‘high’), was used for distributing the sample units for each class. For this purpose, we merged our low and very low classes (1–10%) to ‘low’ in order to enable a visual interpretation of feasible classes. A total of 225 grid-cells per year – 75 within the “undisturbed forest” stratum and 150 within the “logged/burned forest” stratum (50 per disturbance class as suggested by Congalton, 1991), were visually interpreted for each of the 4 years. The visual interpretation was done for grid-cells of 300 m × 300 m. The result accuracy was assessed through error matrices. Although we have selected points only within the forest, eventual errors in the forest mask could lead to some points being interpreted as non-forest.

## 4 Results

We assessed 15-years of forest cover changes within a region highly impacted by deforestation and selective logging. We firstly produced a baseline forest mask for year 2000 with an overall accuracy of 93.7%. This forest mask was updated yearly with new deforestation using the same methodology. Additionally, for each year we produced information on forest disturbance intensity through the grid-cell approach, the accuracy of this information was assessed for selected years as described in Section 3.2. Error matrices comparing the reference data with classified data allowed to assess overall, producer and user accuracies for “undisturbed” forest and different forest disturbance levels (low, medium, high). The overall accuracy considering 4 classes (undisturbed, low, medium, high) is 66%. This accuracy is higher (84%), when the legend is simplified into “undisturbed” and “disturbed” forest. A global error matrix with the information of the 4 years and 4 classes is presented in Table 3a, Table 3b. The main confusion occurs between the classes “undisturbed” and “lightly disturbed”, which is to be expected considering the difficulties in visually interpreting areas of low intensity disturbances, in addition to classification errors. Moreover, confusion also occurs between the intermediate disturbance levels, highlighting the difficulty of validating classes with arbitrary thresholds, and considering that the interpreter did not use information from soil fraction imagery. Nevertheless, it was an attempt to better understand the strengths and limitations of our mapping results and to provide validation information based on an independent dataset.

**Table 3a:** Error matrix including data from year 2003, 2007, 2011 and 2015.

		Reference data					Total	User's accuracy (commission)
		“Undisturbed”	Low	Medium	High	Non-forest		
Classified	“Undisturbed”	236	51	11	2	–	300	78.7%
	Low	59	95	39	7	–	200	47.5%
	Medium	12	15	104	61	8	200	51.5%
	High	4	8	38	149	1	200	74.5%
	Total	310	169	191	219	9		
	Producer's accuracy (omission)	76.1%	56.2%	53.9%	68.0%	Overall accuracy = 65.6%

**Table 3b:** Error matrix considering the binary (undisturbed vs. disturbed) classification – example of year 2007.

		Reference data		Total	User's acc (commission)
		“Undisturbed”	“Disturbed”		
Classified	“Undisturbed”	57	18	75	76.0%
	“Disturbed”	16	134	150	89.3%
	Total	73	152	191	
	Producer's acc (omission)	78.1%	88.2%	Overall accuracy = 84.9%	

### 4.1 Deforestation and logging patterns

**Fig. 7:**
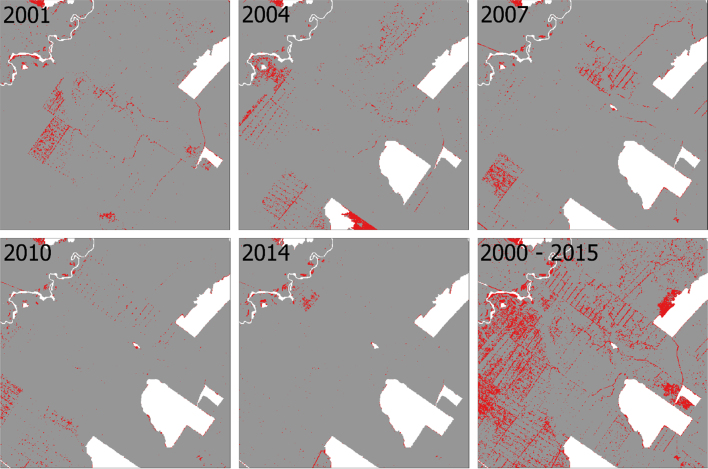
Maps of deforestation (white color) and logging openings (red color) for years 2001, 2004, 2007, 2010, 2014, and the sum of all years over period 2000–2015. (For interpretation of the references to color in this figure legend, the reader is referred to the web version of this article.)

**Fig. 8:**
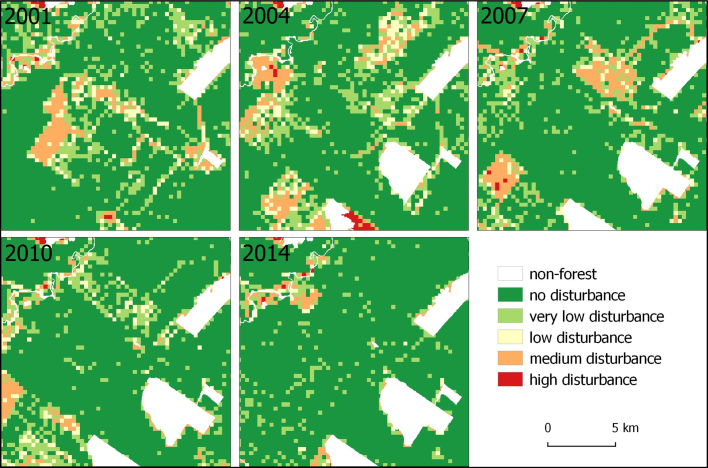
Maps of disturbance intensity for years 2001, 2004, 2007, 2010, and 2014.

The total amount of deforested areas, which represented 18.8% of the total area in 2000, increased to 37.1% in 2015. The areas detected as disturbances within the forest (through the soil fraction) (Fig. 7) represents only 2.3% (minimum) to 6.5% (maximum) of the forest area in year 2006 and year 2000, respectively. Such areas correspond mostly to logging infrastructure (e.g. logging decks, roads) and partially to canopy openings (in case of more disturbed areas). However, the actual area affected by logging activities (disturbed areas) is much higher, considering that the trees can be harvested up to ∼180 m from the logging roads and decks (Souza and Barreto, 2000) or even further (∼350 m), according to Monteiro et al. (2003). From our grid-cell assessment (Fig. 8), we identified that areas affected by logging activities (and in some minor cases by associated fires) annually ranged from 23% (2006) to 39% (2001) of the forest area, considering all forest disturbance intensities. For all years, the areas considered as very low intensity disturbance (1–5%) predominate in relation to the other classes of disturbances. The classes from low to high intensities cover approximately the same area as the very low intensity disturbance areas (Table 4).

**Table 4:** Percentage of the total area for the different land cover classes, considering the grid cells for estimating disturbance.

	**2000**	**2001**	**2002**	**2003**	**2004**
	%	%	%	%	%
Non-forest	18.8%	20.4%	21.3%	24.2%	27.8%
“Undisturbed” forest	54.4%	48.3%	52.4%	49.3%	50.2%
Very low disturbance	11.5%	13.8%	13.2%	13.1%	11.5%
Low disturbance	4.3%	5.5%	5.0%	4.6%	3.8%
Medium disturbance	6.9%	9.2%	6.9%	6.8%	5.3%
High disturbance	4.1%	2.9%	1.1%	2.1%	1.5%

	**2005**	**2006**	**2007**	**2008**	**2009**
	%	%	%	%	%
Non-forest	30.9%	31.6%	32.4%	33.3%	33.9%
“Undisturbed” forest	46.2%	52.3%	46.7%	47.7%	42.9%
Very low disturbance	12.0%	8.9%	11.1%	9.1%	10.1%
Low disturbance	3.9%	2.9%	3.7%	2.9%	3.8%
Medium disturbance	5.5%	3.6%	5.2%	4.7%	6.9%
High disturbance	1.4%	0.6%	0.8%	2.4%	2.3%

	**2010**	**2011**	**2013**	**2014**	**2015**
	%	%	%	%	%
Non-forest	34.1%	34.8%	36.0%	36.4%	37.2%
“Undisturbed” forest	46.1%	44.5%	45.5%	44.5%	45.3%
Very low disturbance	9.9%	9.8%	8.7%	9.1%	8.6%
Low disturbance	3.8%	3.4%	2.9%	3.1%	3.0%
Medium disturbance	5.5%	5.5%	4.8%	5.6%	4.5%
High disturbance	0.7%	1.9%	2.2%	1.3%	1.5%

**Fig. 9:**
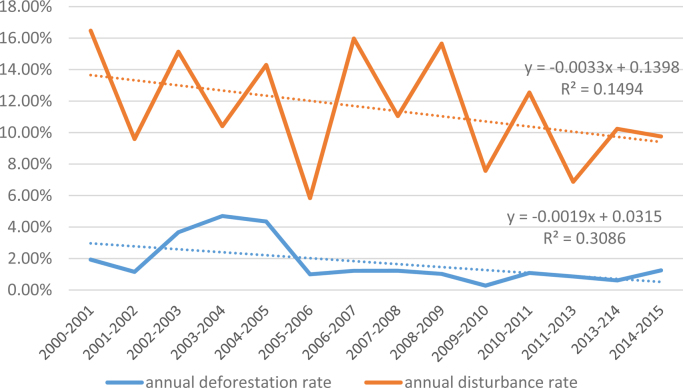
Annual variation of deforestation and degradation rates.

**Fig. 10:**
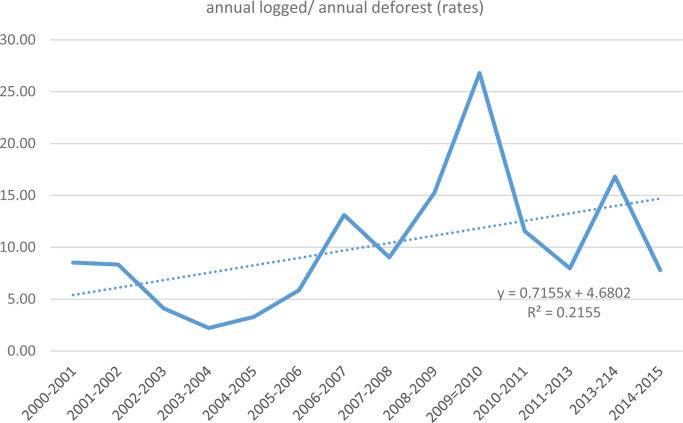
Proportional increase of logging affected areas in relation to deforestation.

The average annual rate of deforestation from 2000 to 2015 is c. 1.7% y^−1^. Annual deforestation rates were highest in the period from 2001 to 2005, then decreased considerably from 2005 onwards. The annual rates of areas affected by disturbances from selective logging are higher than deforestation rates for the whole period of analysis (Fig. 9). In fact, the ratio between annual rates of disturbance and annual rates of deforestation is increasing over time from ca. 5 in 2000 to ca. 15 in 2015 (Fig. 10).

### 4.2 Land cover transitions and recurrence of disturbances

Notably, the spatial–temporal pattern of these disturbances within the forest is very dynamic and logging evidences disappear usually after one or few years. Thus, the areas mapped as logging openings in one year will be quickly recovered and mapped again as “undisturbed” forest in the following years if such an area is not deforested or repeatedly disturbed. A closer look at the changes occurring annually reveal that the areas affected by selective logging were always much larger than the deforested areas (Table 5, Table 6). Moreover, the area of “disturbed” forest, which is later deforested, is more than double the area of “undisturbed” forest being deforested, concerning the whole period of analysis. The change matrices show that the majority of regrowth (changes from “disturbed” to “undisturbed” forest) occurs mainly in areas of low intensity disturbances. However, the low intensity disturbed areas were often detected as a higher intensity in previous years, and thus are relicts from former logging activities.

**Table 5:** Example of change matrix for the year 2000 to 2001 period (area in ha).

**Table 6:** Change trajectories examples for year 2000–2001, 2004–2005, 2009–2010, and 2014–2015 periods.

We also analyzed the temporal trajectory of remaining forest by assessing the number of times that disturbances were identified on forest areas that remained forest until 2015 (i.e. which appeared as not deforested by this date). Only grid-cells containing 100% of forest (disturbed or not) were assessed. In this analysis, we observe that approx. 45% of these forests remaining in 2015 were identified at least once as “disturbed” (considering disturbance levels >5%) during the period 2000–2015. Most of these forests were identified as “disturbed” between one to four times during the 15-years period. However, some limited forest areas were identified as disturbed for a longer period, e.g. in case of occurrence of logging and burning events or due to more persistent features such as roads. Moreover, more than 70% of the deforested areas by 2015 were mapped previously as disturbed forest, based on the grid analysis. From the overall disturbed forest area affected by selective logging up to 2014 (>5% disturbance level), c. 25% had been deforested by 2015.

## 5 Discussion

In this paper, we assess the dynamics of forest cover changes in a study area within the Brazilian Mato Grosso State due to either deforestation (conversion to other land cover) or forest disturbance (mainly by selective logging) over a time period of 15 years (2000–2015). Our results reveal that although the total deforested area is increasing over the years, the amount of forest areas converted to agricultural lands were much smaller compared to the areas disturbed by selective logging. These differences are even more significant if we consider all the areas that have shown some level of disturbance at least once during this period. However, in contrast to deforestation, which normally consist of a permanent or long term change to other land cover types, the areas under selective logging are often followed by forest regeneration in the short term, even though part of these areas will end up being deforested at some stage. Notwithstanding the canopy closure being considerably fast (1–4 years), as highlighted by Asner et al. (2009), the carbon stocks and ecological processes are affected over a much longer period in these forested areas of the Amazon.

Our mapping results are conditioned by the sensor's spatial resolution and face some limitations such as the partial mapping of roads, and the exclusion of some heavily burned areas (classified as deforested in one year) from analysis of the subsequent years. Moreover, small natural gaps in the forest canopy (rock outcrops), occurring in the North of the test area were kept within the forest mask and were identified as highly disturbed. However, these issues have minor impacts on the final results due to their reduced spatial extent. Another aspect of our approach is that it does not allow for the precise delineation of boundaries of areas affected by selective logging, which is also pointed by Souza (2013) about applying a buffer around mapped logging pixels. However, by using fixed grid cells, we provide spatially consistent assessment over time, thus enabling inter-annual comparisons of forest areas affected by disturbance events. Our baseline forest mask achieves 93.7% of overall accuracy, which as higher then PRODES (89.7%) and GFC (86.1%) when compared to the same independent validation dataset.

We assessed the reproducibility of our methodological approach by using forest masks from an independent publicly available dataset: the PRODES dataset (INPE, 2008). Forest/non-forest masks are derived from the PRODES dataset for the same four years we had validation data and combined with the respective soil fraction information (the PRODES products delineates only deforestation patches and not disturbances within the forest). Using the PRODES forest mask, the resulting mean overall accuracy is lower (74.6%) than with our original approach. This is explained by the difference in the forest masks as the disturbance information is obtained with the same approach (soil fraction). For instance, PRODES maps larger deforestation patches (>6.5 ha) and consequently misses small spatial details. Moreover, roads and water bodies are not considered in PRODES dataset, and a geolocation shift can cause localized problems. However, the use of PRODES shows that our approach can be applied with other forest masks.

INPE has mapped forest degradation through the DEGRAD project (INPE, 2008). Despite the differences in methodologies and definitions, a comparison of our results with DEGRAD, show that 75% and 80% of the areas identified by the DEGRAD as degraded forest areas in 2007 and 2011 respectively were also identified in our study as “disturbed forest areas”. In 2011, most these areas corresponded to the moderate or high disturbance classes in our study. However, we also found that many areas under a process of selective logging are not mapped by DEGRAD. In addition to DEGRAD, INPE runs project called DETEX, which is focused on mapping selective logging in the Brazilian Amazon. However, we could not use this data, as the results from DETEX are not available publicly.

Hansen et al., 2013, Hansen et al., 2013b report that their Global Forest Change (GFC) product does not map or measure forest degradation characterized by a decrease of tree crown cover within a Landsat pixel unless it leads to a non-forest state. Thus, the pixels identified as tree cover loss in the GFC product include pixels affected by logging activities, such as large-enough (>1 Landsat pixel) forest canopy gaps, logging decks or logging roads, with a full removal of the tree canopy within such pixels. Our estimate of total disturbed area from 2001 to 2014 (accumulated area) is compared to that of GFC tree cover loss between 2001 and 2014 over the 2015 forest area (using our mask). This allows to assess the additional information provided by this (sub-pixel) soil fraction approach compared to full tree cover removal approach of the GFC product. We also quantify the proportion between full crown cover removal and partial crown cover removal due to selective logging activities. Our accumulated disturbance area represents 19% of the forest area by 2015, when the tree cover loss area from GCF for the same period, represents only 4.2% of the 2015 forest area. The full crown cover removal area represents 22% of the partial crown cover removal area. This demonstrates the importance of using sub-pixel information for the assessment of selective logging with remote sensing imagery. Tyukavina et al. (2017) used the GFC dataset as stratification layer for a study on forest disturbances in the Brazilian Legal Amazon. However, the lack of partial crown cover removal information in the GFC data set leads most probably to a significant underestimation of logging-related disturbances in this study.

We also compare our results with results from Asner et al., 2005, Asner et al., 2013 who assessed selective logging in Mato Grosso for the period 2000 to 2002. The dataset consists in binary information: logged forest/non-logged forest. For comparison, we apply our grid approach to the Asner dataset (counting the number of logged pixels within grid-cell) and compare the result with our product, reclassified as disturbed/non-disturbed. From this analysis, we find that from the total logged area identified by Asner et al. for year 2000 (corresponding to 19% of the forest area), only 64% are identified as disturbed area in our product. Conversely from our total logged area for same year (corresponding to 27% of the total forest area) only 44% were identified as disturbed area in Asner et al., indicating spatial discrepancies between the two datasets. Based on a visual inspection, we found a significant number of places with visible logging evidence in the image used in our study without identification in Asner's dataset. These divergences are partly due to the differences in the satellite image acquisition dates but also related to the differences in approaches (e.g. selected thresholds and single date analysis versus image pairs).

Regarding the succession of deforestation following forest disturbances, we observe that the area of “disturbed forest” converted to deforested areas is considerably higher than the conversion from “undisturbed” forest (Table 6). Considering the overall period 2000–2014 and the deforested areas in 2015, our results show that at least 30% of the deforestation in 2015 had been mapped as “forest disturbance pixels” before based on soil fraction images. This percentage is much higher (>70%) if we consider the total disturbed area assessed by the grid-cell approach.

From the total disturbed forest from 2000–2014, c. 25% had been deforested by 2015. Stone and Lefebvre (1998), using images acquired 5 years apart (1986 and 1991), showed that 91% of the selectively logged forest in 1986 were classified as forest again in 1991 and only 9% were converted to pastures. This can be related to the results of Matricardi et al. (2005) who showed that 17% of the logged forests detected during the period of 1992–2001, were deforested by 2002. Asner et al. (2006) in a broader study reported also a higher probability of deforestation for logged areas. They assessed that 16% of selectively logged forest between 1999 and 2004 were deforested by 2005. For a similar period (2000–2004), we estimate that 14.6% of the total disturbed area was converted to non-forest by 2005 (considering only grid cells with 100% forest).

We also show that the annual newly disturbed forest areas are much larger than the annual deforested areas. However, only a portion of such areas continues to appear as disturbed for several years. If the disturbances were mapped from images with a longer time interval (more than 3–4 years apart) much less disturbance would be identified.

Compared to other methods, our proposed approach gives the possibility of not only mapping forest disturbances but also assessing them consistently through time, as the used grid-cells (300 m × 300 m) remain stable. This emerged as an important aspect during the methodology development as selective logging is a very dynamic process in time and space. Moreover, our method relies in a simplified classification approach for building and updating a forest mask, thus allowing to focus the analysis on the forested areas for each year. This method could be adapted to the use of existing data, like e.g. the PRODES forest mask, or to the application in other geographic areas. The combination of soil fraction images (sub-pixel analysis) with an object-based classification approach was a key to maximize the contribution of each method for (1) delineating forest/non-forest patches and keeping the areas of selective logging within the forest polygons, and (2) highlighting the contribution of the soil component related to disturbances within the forest. The object-based approach provides a more precise delineation of forest and non-forest areas by segmenting the image into meaningful objects closely related to real word objects (Blaschke, 2010). Moreover, this approach is improving the classification accuracy and allowing the integration of expert knowledge into the classification process (Platt and Rapoza, 2008). This result can be used independently for the reporting on deforestation. The soil fraction data derived from the “unmixing approach”, focused on forest areas, allows to retrieve detailed information from Landsat imagery on the mapping of selective logging, which is an important step when regarding the limitation of the medium spatial resolution of Landsat. And finally, the combination of the results from by both approaches on a grid-cell basis enable the assessment of the extent of logging impacts, logging intensity and its dynamics over time. The main strength of the grid-cell approach is to allow keeping a spatial consistency over time for the assessment these forest cover disturbances, which are highly dynamic in the southern Brazilian Amazon region.

## 6 Conclusion

We assessed annual forest disturbances, with a focus on logging activities, in a test area of Mato Grosso State within a time period of 15 years. Our test area has been subject to timber exploitation for a long time and can be considered representative of many other areas in the region.

Our method provides a feasible means of assessing forest disturbances consistently over time using medium resolution Landsat satellite images, and allows to assess deforestation and forest disturbances due to selective logging. For this, building a forest mask from an object-based classification approach was an essential step in order to distinguish between cleared areas (for agriculture or other lands) from small openings related to selective logging. The use of fraction images derived from Landsat imagery is also an essential step to identify logging openings, as it allows highlighting features at a subpixel level. Finally, the use of this information in grid-cell approach, allows the assessment of forest disturbances over time providing estimates of the impacted areas. The method can be adapted to other regions e.g. by using different grid sizes or by incorporating other processes or other types of information. It is intended to apply the method to larger regions within the Amazon basin and to adapt it to finer resolution imagery such as data from the Sentinel-2 satellite at 10 m × 10 m spatial resolution, which is available more extensively since mid-2016.
